# V*γ*4+ T Cells: A Novel IL-17-Producing *γδ* T Subsets during the Early Phase of Chlamydial Airway Infection in Mice

**DOI:** 10.1155/2018/6265746

**Published:** 2018-02-18

**Authors:** Li-da Sun, Sai Qiao, Yue Wang, Gao-ju Pang, Xiao-yu Zha, Teng-Li Liu, Hui-Li Zhao, Ju-You Liang, Ning-bo Zheng, Lu Tan, Hong Zhang, Hong Bai

**Affiliations:** Department of Immunology, School of Basic Medical Sciences, Tianjin Medical University, Tianjin Key Laboratory of Cellular and Molecular Immunology, Key Laboratory of Educational Ministry of China, Tianjin 300070, China

## Abstract

Our previous studies showed that *γδ* T cells provided immune protection against Chlamydial *muridarum* (Cm), an obligate intracellular strain of chlamydia trachomatis, lung infection by producing abundant IL-17. In this study, we investigated the proliferation and activation of lung *γδ* T cell subsets, specifically the IL-17 and IFN*γ* production by them following Cm lung infection. Our results found that five *γδ* T cell subsets, V*γ*1+ T, V*γ*2+ T, V*γ*4+ T, V*γ*5+ T, and V*γ*6+ T, expressed in lungs of naïve mice, while Cm lung infection mainly induced the proliferation and activation of V*γ*4+ T cells at day 3 p.i., following V*γ*1+ T cells at day 7 p.i. Cytokine detection showed that Cm lung infection induced IFN*γ* secretion firstly by V*γ*4+ T cells at very early stage (day 3) and changed to V*γ*1+ T cells at midstage (day 7). Furthermore, V*γ*4+ T cell is the main *γδ* T cell subset that secretes IL-17 at the very early stage of Cm lung infection and V*γ*1+ T cell did not secrete IL-17 during the infection. These findings provide in vivo evidence that V*γ*4+T cells are the major IL-17 and IFN*γ*-producing *γδ* T cell subsets at the early period of Cm lung infection.

## 1. Introduction

Chlamydia, an obligated intracellular bacterium, can cause various human diseases by the two chlamydial species, Chlamydia *trachomatis* and Chlamydia *pneumoniae*. C. *pneumoniae* causes respiratory diseases like bronchitis, sinusitis, and pneumonia, whereas C. *trachomatis* is a major cause of ocular and sexually transmitted diseases [1]. The mouse pneumonitis strain of C. *trachomatis*, recently designated as Chlamydia *muridarum* (Cm), has been widely used in mouse models of respiratory and genital tract infections [2]. Th1 response has been demonstrated to be the dominant protective determinant for controlling chlamydial infection in human and mouse models [3–5]. More recently, our and others' studies indicate that Th17 plays an important role in host defense against chlamydial infection through either promoting Th1-type cell responses or working synergistically with IFN*γ* [6]. Therefore, the development of both Th1 and Th17 cell immune responses is optimal for host defense against chlamydial lung infections.

Although *αβ*T cells dominate Ag-specific effector and memory stages, *γδ* T cells have fused adaptive and innate-like qualities to be at the forefront of immune responses. *γδ* T cells can directly kill infected cells, produce molecules required for pathogen clearance, and release immunomodulatory cytokines such as IFN*γ*, IL-17, and IL-4 [7, 8] with no MHC-limited recognition and antigen processing or presentation [9–11]. A number of recent studies using various experimental mouse models have shown that *γδ* T cell is also a major producer of IL-17 following intracellular pathogen infections, including H1N1 influenza virus [12], *Staphylococcus aureus* [13], *Listeria monocytogenes* [14], and Salmonella enterica enteritidis [15]. In general, activated *γδ* T cells mainly make resistance to pathogens by secreting IFN*γ*. However, a growing number of studies recently showed that *γδ* T cells are an important source of proinflammatory cytokine IL-17 [16], and in some researches, IL-17-producing *γδ* T cells expanded more faster than *αβ*T cells and worked more effectively than adaptive CD4+ Th17 cells [17, 18].

According to the difference of TCR*γ*, the mouse *γδ* T cells are divided into 6 kinds of *γδ* T cell subsets, including V*γ*1+ T, V*γ*2+ T, V*γ*4+ T, V*γ*5+ T, V*γ*6+ T, and V*γ*7+ T cells and lung *γδ* T cells of naïve mice predominantly comprising V*γ*1+ and V*γ*4+ subsets [19, 20]. Study on a variety of disease models showed that the specific TCR-expressing V*γ*T cells play its unique function [21]. For example, V*γ*1+ T cells aggravated airway responsiveness, whereas V*γ*4+ T cells reversed airway responsiveness [22]. Although the function of *γδ* T cells has been demonstrated in a variety of mouse models such as Klebsiella pneumonia [23] and cryptococcal pneumonia [24], the subsets of *γδ* T cells in lung inflammation were seldom investigated. Current studies have shown that V*γ*4+ T cells are the dominant IL-17-producing cells in infectious or noninfectious diseases. The ability of V*γ*1+ *γδ* T cells to produce IFN*γ* was significantly reduced in the late phase of blood-stage Plasmodium berghei XAT (PbXAT) parasite infection [25]. In infectious model of Lester coli [26], *Escherichia coli* [27], Bacillus subtilis [28], and V*γ*4+ T quickly secreted a large number of IL-17 combined with IL-23 produced by DC during infection. V*γ*4+ T cells produced IL-17 but not IFN*γ* in a mouse model of collagen-induced arthritis (CIA) [29].

Our previous study found that depletion of *γδ* T cells reduced IL-1*α* production by dendritic cells, which was associated with a reduced Th17 protective response during Cm infection [6]. Large amounts of IFN*γ* and IL-17 existed at the early stage of infection participate in host immune response against Chlamydia infection. However, the sources of IFN*γ* and IL-17 production by which of *γδ* T cell subset in lungs and their biological activities following chlamydial infection remained unclear. Here, we will further elucidate the properties and the role of *γδ* T cell subsets during Cm lung infection and also provide a theoretical basis for clinical diagnosis and treatment of chlamydia infectious diseases and their complications.

## 2. Materials and Methods

### 2.1. Mice and Microorganisms

Breeding pairs of TCR*δ*−/− mice (C57BL/6) were gifted from Nankai University, Professor Yin Zhinan. The WT control mice (C57BL/6) were purchased from Laboratory Animal Center, Academy of Military Medical Sciences. Mice were housed in specific pathogen-free conditions in Tianjin Medical University with autoclaved cage, food and water, and filtered airflow. Age- and sex-matched mice at 6–8 weeks old were used for study. Chlamydial muridarum (Cm), a mouse chlamydial strain, was reproduced and purified as previously described [30]. Briefly, Cm was grown in HeLa-229 cells in DMEM medium containing 10% fetal bovine serum (FBS) and 2 mM glutamine. Elementary bodies (EBs) were purified by discontinuous density gradient centrifugation. Titers of EBs were determined by measuring inclusion-forming units (IFUs) after immunostaining, and aliquots of the EB stock were stored at −80°C.

### 2.2. Infection of Mice and Quantification of Lung Chlamydial Loads

Mice were sedated with isoflurane and infected intranasally with 1 × 10^3^ IFUs of C. muridarum in 40 *μ*l sucrose-phosphate-glutamic acid (SPG) buffer. Mouse body weights were monitored daily. Mice were euthanized at the indicated time points, and the lungs were aseptically isolated and homogenized using a cell grinder in SPG buffer. The tissue homogenates were centrifuged, and supernatant was stored at −80°C until being tested. The bacterial loads in lungs at day 3, day 7, and day 14 after Cm infection were titrated by infection of HeLa cell monolayers as previously described [31].

### 2.3. Lung Mononuclear Cell Preparation

Lung mononuclear cells were prepared as described previously [9]. Briefly, the lung tissues were incubated in digestive buffer (containing 100 *μ*g/ml DNase [Sigma-Aldrich] and 2 mg/ml collagenase type XI [Sigma-Aldrich, St. Louis, MO, USA]) for 60 min at 37°C and added 2 mM EDTA 5 min before incubation finished. Then the cell population was purified by mixing with 35% Percoll (Sigma-Aldrich) and centrifuged for 20 min at 750 g, followed by lysis of erythrocytes with ammonium chloride-potassium (ACK) lysis buffer (150 mmol/l NH_4_Cl, 10 mmol/l KHCO_3_, and 0.1 mmol/l EDTA). The cells were washed twice using RPMI 1640 with 2% fetal calf serum and resuspended in complete RPMI 1640 medium (containing 10% FBS) for further experiment.

### 2.4. Reverse Transcriptase-Polymerase Chain Reaction (RT-PCR)

To analyze the expressions of TCR V*γ* transcripts, total RNA was extracted from frozen lung tissues using Trizol agent (Invitrogen) according to the manufacturer's instruction. The isolated total RNA was reversely transcribed into cDNA (TaKaRa). Special primers for V*γ*1, V*γ*2, V*γ*4, V*γ*5, V*γ*6, and V*γ*7 were used to amplify cDNA. And *β*-actin, a housekeeping gene, was used as a control. The primers used in the PCR analysis were as follows: V*γ*1 (320 bp), forward: 5′-ACACAGCTATACATTGGTAC-3′, reverse: 5′-CTTATGGAGATTTGTTTCAGC-3′; V*γ*2 (270 bp), forward: 5′-CGGCAAAAAACAAATCAACAG-3′, reverse: 5′-CTTATGGAGATTTGTTTCAGC-3′; V*γ*4 (310 bp), forward: 5′-TGTCCTTGCAACCCCTACCC-3′, reverse: 5′-CTTATGGAGATTTGTTTCAGC-3′; V*γ*5 (300 bp), forward: 5′-TGTGCACTGGTACCAACTGA-3′, reverse: 5′-CTTATGGAGATTTGTTTCAGC-3′; V*γ*6 (300 bp), forward: 5′-TGTGCACTGGTACCAACTGA-3′, reverse: 5′-CTTATGGAGATTTGTTTCAGC-3′; V*γ*7 (380 bp), forward: 5′-AAGCTAGAGGGGTCCTCTGC-3′, reverse: 5′-CTTATGGAGATTTGTTTCAGC-3′; *β*-actin (582 bp), forward: 5′-CTTATGGAGATTTGTTTCAGC-3′, reverse: 5′-ATGAGGTAGTCMGTCAGGT-3′. The products were electrophoresed in 1% agarose gel containing Gel-Red (0.01%). The bands were visualized and photographed by automatic gel imaging system and were analyzed for density on Image J software.

### 2.5. Flow Cytometry

Lung mononuclear cells were aseptically prepared from mice at different time points postinfection and incubated with anti-CD3, anti-TCR*γδ*, anti-CD69, anti-TCRV*γ*1, anti-TCRV*γ*4, and isotype control Abs for 30 min on ice for surface marker analysis. For intracellular cytokine analysis, single cell suspensions were stimulated with PMA (50 ng/ml)/ionomycin (1 *μ*g/ml) (Sigma) for 6 hours at 37°C in the presence of 20 mg/ml brefeldin A (Sigma). After the stimulation, cells were washed with FACS buffer twice and incubated with Fc receptor (FcR) block antibodies (anti-CD16/CD32; eBioscience) for 15 min on ice to block nonspecific staining. Surface markers (CD3, TCR*γδ*, TCRV*γ*1, and TCRV*γ*4) were stained first. The cells were then fixed with 4% *w*/*v* paraformaldehyde in PBS and permeabilized with permeabilization buffer (0.1% saponin [Sigma] Sigma, 2% heat-inactivated FCS, and 0.1% NaN_3_ in PBS), subsequently stained with anti-IFN*γ*, IL-17, or corresponding isotype control Abs (eBioscience). The raw data were collected using FACS CantoII flow cytometer (BD Biosciences) and were analyzed using Flowjo 6.0 software.

### 2.6. Statistical Analysis

One-way analysis of variance (ANOVA) and unpaired *t*-test were used to determine statistical significance among groups. IFUs of Cm were converted to logarithmic values and analyzed using ANOVA. The value of *p* < 0.05 was considered as a statistically significant difference.

## 3. Results

### 3.1. *γδ* T Cells Mediated Immune Protection against Cm Infection by Expansion, Activation, and Secreting IFN*γ* and IL-17

**Figure 1:**
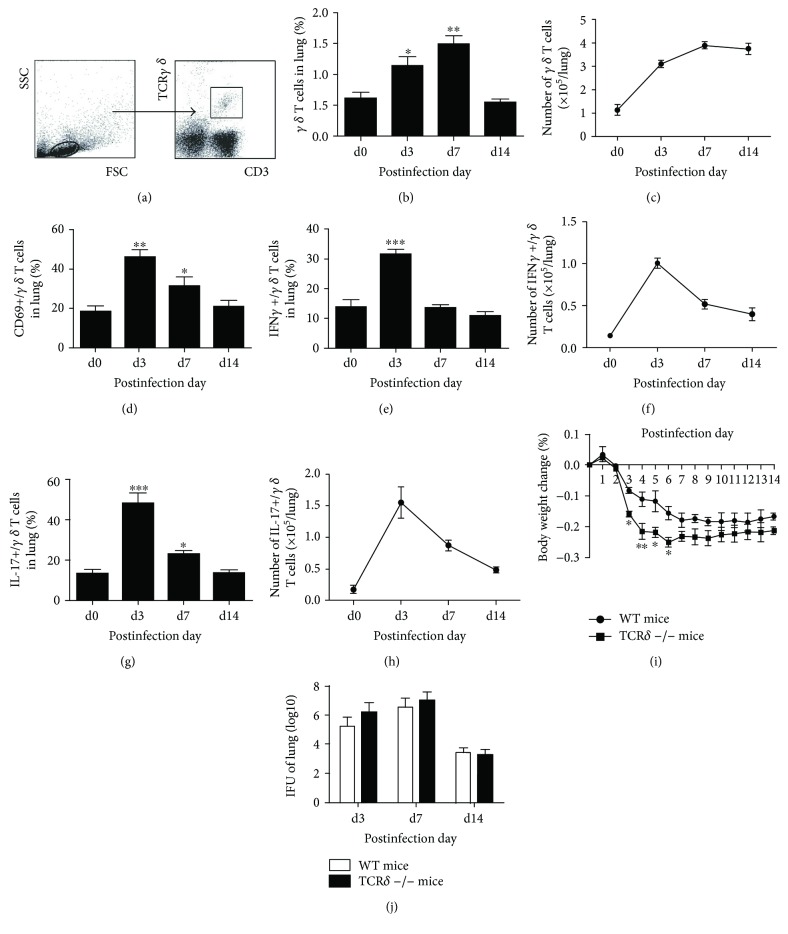
*γδ* T cells provided immune protection against Cm infection by expansion, activation, and secreting *IFNγ* and *IL-17*. The mononuclear cells from WT mice (four/group) killed at specific time points following C. m*uridarum* infection (1 × 10^3^ IFUs) were extracted from the lungs. In gated lymphocytes (a), percentage (b), and absolute number (c) of CD3+ TCR*γδ*+ T cells, expression level of CD69 on CD3+ T CR*γδ*+ T cells (d), percentage (e, g), and absolute number (f, h) of IFN*γ*/IL-17-produing *γδ* T cells were analyzed and calculated by flow cytometry. WT and TCR*δ*^−/−^ mice (four/group) were infected intranasally with C. *muridarum* (1 × 10^3^ IFUs). Body weight changes (i) were monitored daily, and pulmonary C. *muridarum* (j) were assessed at day 3, day 7, and day 14 p.i. as mentioned in Materials and Methods. Shown are the representative data of two independent experiments with similar results presented as mean ± SD. ^∗^*p* < 0.05, ^∗∗^*p* < 0.01, and ^∗∗∗^*p* < 0.001.

*γδ* T cells are the vital components of the innate immune system and play important roles in the early responses to pathogens. Our previous studies have shown that *γδ* T cells are the major producer of IL-17A in the very early stages of infection and depletion of *γδ* T cells by administration of mAb (GL3) against TCR*γδ* i.n. exists more body weight loss following Cm lung infection. The results here keep consistent with our previous studies that the percentage and absolute number of lung *γδ* T cells significantly increased at day 3 postinfection (p.i.) and reached to the highest level at day 7 p.i. Even though the percentage of *γδ* T cells reduced to baseline levels, the absolute number of *γδ* T cells still kept in a relatively higher level (Figures 1(b) and 1(c)). CD69 was generally used for indicating the activation of *γδ* T cells.  Figure 1(d) showed that Cm infection induced *γδ* T cell activation in lungs by increased CD69 expression on *γδ* T cells following Cm infection. Following activation, IFN*γ* or IL-17 secretion by *γδ* T cells was significantly increased especially on day 3 p.i. (Figures 1(e)–1(h)). TCR*δ*−/− mice were used for further confirmation of the function of *γδ* T cells during Cm lung infection in the current researches. With Cm lung infection, TCR*δ*−/− mice had more weight loss compared with WT mice, especiallly at day 3 to 6 p.i. (Figure 1(i)); however, the lung bacterial loads (IFUs) between TCR*δ*−/− and WT mice did not show a significant difference (Figure 1(j)). Furthermore, the lung tissues of TCR*δ*−/− mice had more inflammatory cell infiltration compared with WT mice after chlamydial lung infection (data not shown). All these results implicated that *γδ* T cells contribute to the IFN*γ* and IL-17 production and reduce morbidity during Cm infection, but its role in bacterial clearance is rather limited.

### 3.2. V*γ*1+ T and V*γ*4+ T Cells Are the Major Proliferative Cell Subsets of *γδ* T Cell during Cm Lung Infection in Mice

**Figure 2:**
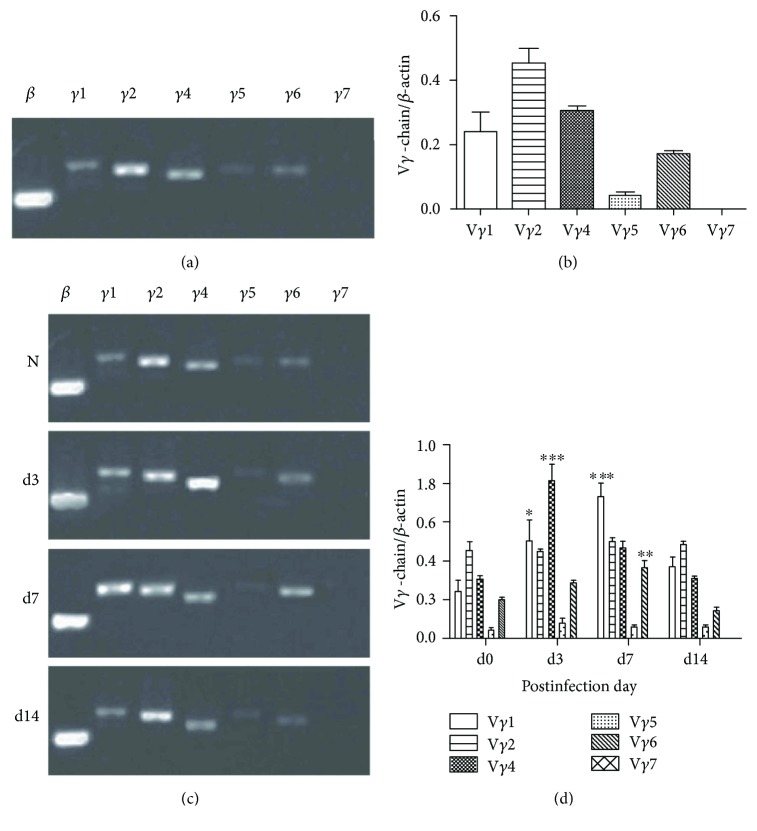
V*γ*1+ T and V*γ*4+ T cell are two main subsets of *γδ* T cells during Cm respiratory tract infection. The types of *γδ* T cell subsets from lung tissues in naïve (a, b) and infected mice (c, d) were defined according to the expression of TCRV*γ* mRNA in lungs detected by RT-PCR. Shown are the representative data of two independent experiments with similar results presented as mean ± SD. ^∗^*p* < 0.05, ^∗∗^*p* < 0.01, and ^∗∗∗^*p* < 0.001.

*γδ* T cells are heterogeneous population that can be subdivided based on the expression of specific V*γ* and V*δ* TCR chains. Although we already demonstrated the importance of *γδ* T cell in the early protection against Cm lung infection, this did not prove that *γδ* T cell subpopulation actually contributes to the *γδ* T cell-mediated early protection. To investigate this, we first analyzed the *γδ* T cell subsets in lungs of naive mice. Our results by using RT-PCR detection showed that there are more than five subpopulations, V*γ*1+ T, V*γ*2+ T, V*γ*4+ T, V*γ*5+ T, and V*γ*6+ T but not V*γ*7+ T cells; in lungs of naive mice, the expression intensity of mRNA is V*γ*2 > V*γ*4 > V*γ*1 > V*γ*6 > V*γ*5 (Figures 2(a) and 2(b)). Next, we further detected the mRNA expression of *γδ* T cell subsets in the lungs at different time point post-Cm infection. The results showed that TCRV*γ*4 was significantly upregulated at day 3 p.i. while TCRV*γ*1 mRNA expression was significantly increased at day 7 p.i (Figures 2(c) and 2(d)). V*γ*6+ mRNA also showed a relatively high expression level at day 7 p.i. Collectively, these results showed that V*γ*1+ T and V*γ*4+ T cells are the major proliferative cell subsets of *γδ* T cell in lungs of mice during Cm infection.

### 3.3. Cm Infection Induced Dramatic Proliferation and Activation of V*γ*1+ T and V*γ*4+ T Cells in Lungs

**Figure 3:**
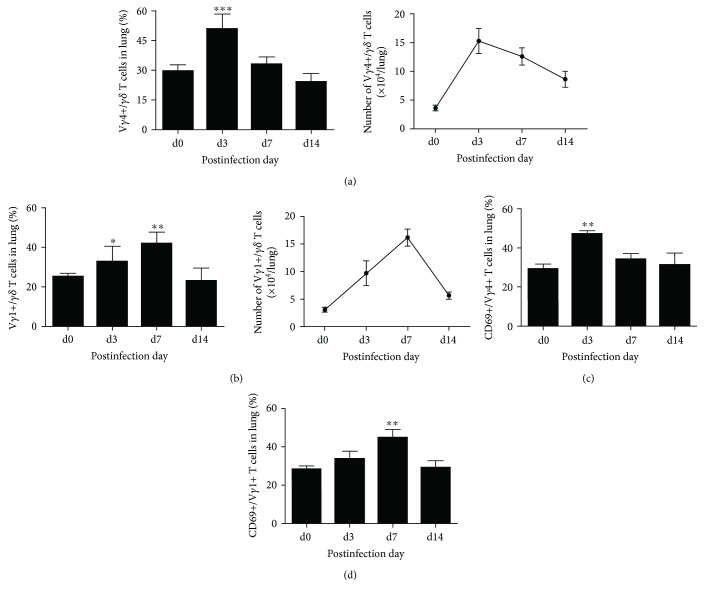
V*γ*1+ T and V*γ*4+ T cell proliferated and activated during Cm respiratory infection. Mononuclear cells in lung tissues at different time points postinfection were extracted. Staining with anti-mouse CD3, TCR*γδ*, TCRV*γ*1, and TCRV*γ*4 antibody to analyze the percentage and absolute number of TCRV*γ*1+ TCR*γδ*+ T cells (a) and TCRV*γ*4^+^TCR*γδ*^+^T cells (b) by flow cytometry. The activation extent of V*γ*1+ T (c) and V*γ*4+ T (d) cell was measured by the expression of CD69, staining with anti-mouse CD69 antibody. The results are presented as mean ± SD. ^∗^*p* < 0.05, ^∗∗^*p* < 0.01, and ^∗∗∗^*p* < 0.001.

Some studies have shown that V*γ*1+ T and V*γ*4+ T cells were induced to proliferate and activate and provide different roles in host defense against pathogen infection. To further confirm the proliferation and activation of the TCR V*γ*1+ and TCR V*γ*4+ *γδ* T cells at an early stage of Cm infection, we examined the lung TCR V*γ*1+ and TCR V*γ*4+ *γδ* T cell percentage and CD69 expression by FACS. As shown in Figure 3, the percentage, absolute number (Figure 3(a)), and CD69 expression (Figure 3(c)) of V*γ*4+ T cell in lungs quickly reached the peak at day 3 p.i. and kept a high level in absolute number at day 7 p.i., while the percentage, absolute number (Figure 3(b)), and CD69 expression (Figure 3(d)) of V*γ*1+ T cells significantly increased at day 3 p.i. and reached the peak at day 7 p.i. Taking these results together, we concluded that Cm infection induced dramatic proliferation and activation of TCR V*γ*4+ and V*γ*1+ *γδ* T cells in the lungs at an early stage.

### 3.4. Both V*γ*1+ and V*γ*4+ T Cells Are the IFN*γ*-Producing γ*δ* T Cell Subpopulations at Different Stages of Cm Infection

**Figure 4:**
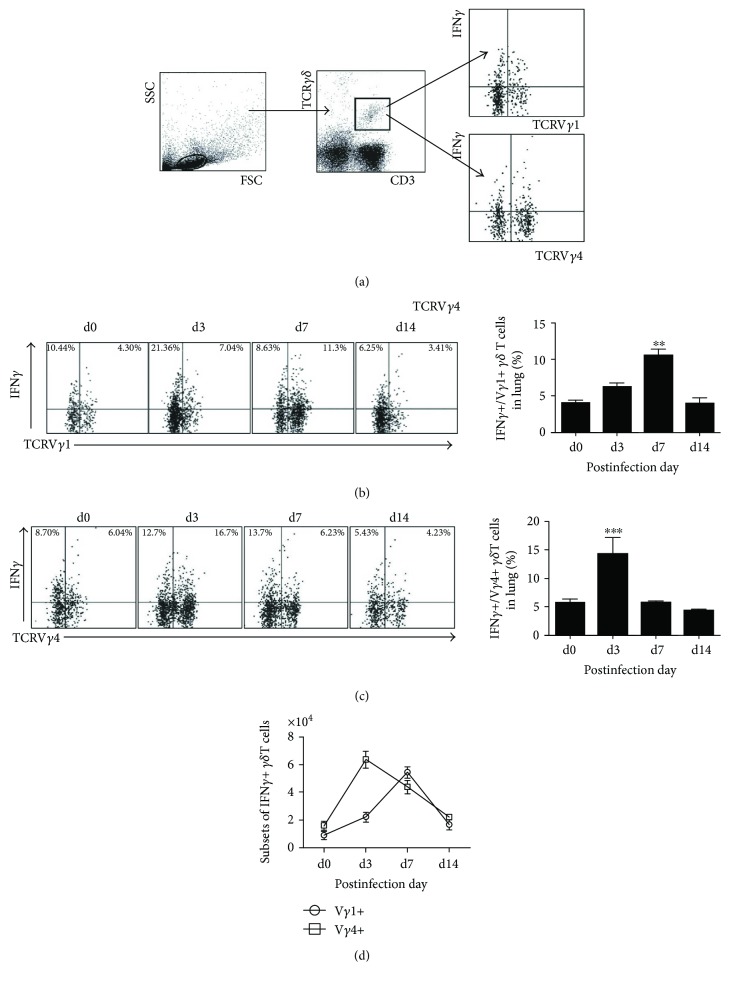
V*γ*4 cells at day 3 p.i. and V*γ*1 cells at day 7 p.i. are the major sources of IFN*γ* during Cm lung infection. IFN*γ*+ V*γ*1+/V*γ*4+ T cells were gated (a). Staining with anti-mouse CD3, TCR*γδ*, TCRV*γ*1/V*γ*4, and IFN*γ*/IL-17 antibody to analyze the percentage and absolute number of IFN*γ*+ V*γ*1+ T cells (b) and IFN*γ*+ V*γ*4+ T cells (c) in lung tissues after Cm infection by flow cytometry. Comparison between IFN*γ*+ V*γ*1+ cell and IFN*γ*+ V*γ*4+ cell with its absolute number (d). The results are presented as mean ± SD. ^∗∗^*p* < 0.01 and ^∗∗∗^*p* < 0.001.

IFN*γ* has been reported to be produced by several different *γδ*+ T cell subpopulations in different stages of disease and mediated various immune functions. As shown in Figure 4, both V*γ*1+ and V*γ*4+ T cells can produce IFN*γ* during Cm lung infection; however, V*γ*4 T cells are the major sources of IFN*γ* at very early time p.i. (day 3) while V*γ*1 T cells at midstage p.i. (day 7) (Figures 4(c) and 4(b)).

After Cm infection, the secretion of IFN*γ* was gradually increased and reached the peak at day 7 p.i. which had significant difference with uninfected group then declined to the basic level at day 14 p.i. (Figure 4(b)). However, the percentage of IFN*γ*+ V*γ*4+ T cells increased rapidly after infection and even reached the peak at day 3 p.i. then restored to the basic level at day 7 p.i. (Figure 4(c)). The absolute number of V*γ*1+ T and V*γ*4+ T cells in lungs (Figure 4(d)) also indicated the similar variation with their percentages. Taking these results together, we concluded that Cm lung infection induces IFN*γ* secretion from V*γ*4+ T cells at very early stage and V*γ*1+ T cells at midstage of infection.

### 3.5. V*γ*4+ T Cells Are the IL-17-Producing *γδ* T Cell Subpopulations at the Very Early Stage of Cm Infection

**Figure 5:**
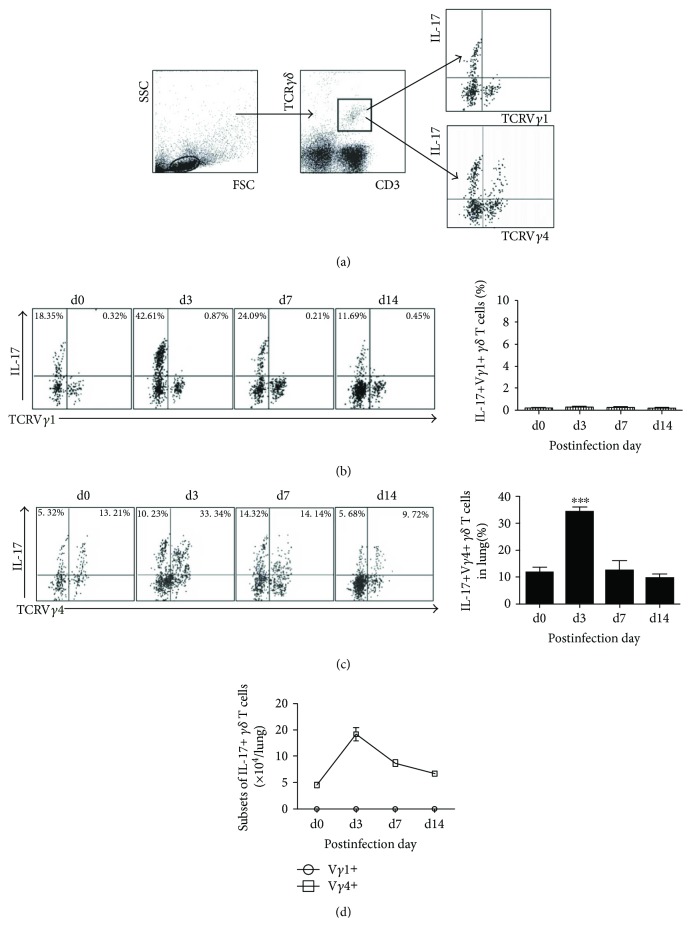
V*γ*4 cells at day 3 p.i. are the major sources of IL-17 during Cm lung infection. IL-17+ V*γ*4+/V*γ*1+ T cells were gated (a), stained with anti-mouse CD3, TCR*γδ*, TCRV*γ*4, and IFN*γ*/IL-17 antibody to analyze percentage of Il-17+ V*γ*1+ T cells (b) and IL-17+ V*γ*4+ T cells (c) in lung tissues after Cm infection. Comparison between IL-17+ V*γ*1+ cell and IL-17+ V*γ*4+ cell with its absolute number (d). The results are presented as mean ± SD. ^∗∗∗^*p* < 0.001.

We further identified IL-17-producing *γδ* T cell subpopulations at different stage of Cm infection by intracellular cytokine staining. Few IL-17+ V*γ*1+ T cells were detected in uninfected mice and had no significant increase following Cm lung infection (Figure 5(b)), whereas V*γ*4+ T cells can secrete large quantity of IL-17 (Figure 5(c)) during Cm lung infection in mice. It was noted that the percentage of IL-17+ V*γ*4+ T cells increased rapidly after infection and even reached the peak at day 3 p.i. and then quickly restored to the basic level at day 7 p.i. All these above results demonstrated that lung Cm-infected V*γ*4+ T cell is the main *γδ* T cell subset secreting IL-17 at the very early stage of Cm lung infection. Meanwhile, there are still a small number of IL-17-producing-V*γ*4-*γδ* T cell subsets which is not identified during Cm infection, which should be discussed further.

## 4. Discussion

*γδ* T cells provide immune protective in Chlamydia trachomatis infection. Here, we demonstrate the coincident involvement of multiple *γδ* T cell subsets. While a significant proportion of naive lung *γδ* T cells exhibited an activated phenotype, activation was clearly enhanced in infected mice, most notably in respect to V*γ*1 and V*γ*4 expression. Based on the kinetics of IFN*γ* and IL-17 production by *γδ* T cells, we tested the function of *γδ* T cell subset in the ensuing immune response against Cm infection. Surprisingly, we demonstrated that V*γ*4+ T cells are the major IL-17 and IFN*γ*-producing *γδ* T cell subsets at the early period of Cm lung infection, while V*γ*1+ T cells are responsible for the secretion of IFN*γ* at midstage.

*γδ* T cells express a distinct TCR composed of the TCR*γ*- and *δ*-chains [32]. Human *γδ* T cells can be divided into three main populations based on *δ* chain expression: V*δ*1, V*δ*2, and V*δ*3 *γδ* T cells. *γδ* T cells in lungs of infected mice are classified into six subsets, V*γ*1+ T, V*γ*2+ T, V*γ*4+ T, V*γ*5+ T, V*γ*6+, and V*γ*7+ *γδ* T cells in local responses to Streptococcus pneumoniae infection [33]. In our present study, according to distinct TCR *γ* chain expression, there are five subpopulations, V*γ*1+ T, V*γ*2+ T, V*γ*4+ T, V*γ*5+ T, and V*γ*6+ T but not V*γ*7+ T cells in lungs of naive mice. It was reported [34] that lung V*γ*1+ and V*γ*4+ *γδ* T cells proliferated significantly in pulmonary Mycobacterium bovis bacille Calmette-Guerin infection, and study on sepsis [35] showed that V*γ*1+ *γδ* T cells preferentially expanded over time after infection with PbXAT parasites. Similarly, our results showed that the increase of CD69+ V*γ*4+ T cells and CD69+ V*γ*1*δ*T cells showed to be concordant with subpopulation proliferation and infected lung *γδ* T cells comprising predominantly V*γ*1+ and V*γ*4+ subsets.

The effector functions of *γδ* T cells can be broadly classified by their tissue localization, status of activation, and expression of TCR variable genes [36]. IL-17-producing *γδ* T cells play a crucial role in innate immunity against various infections [26, 36, 37]. Our previous study has shown that *γδ* T cells are the major producer of IL-17 in the very early stages of infection, and the depletion of *γδ* T cells by administration of mAb (GL3) against TCR*γδ* i.n. exists more body weight loss following Cm lung infection, which suggested that *γδ* T cells played a protective role in mice Chlamydia lung infection [16]. These results are in accordance with our data using TCR*δ*−/− mice in this paper. It is worth mentioning that *γδ* T cell is the highest producer of IL-17A but the protection conferred by IL-17A is mainly mediated by Th17 cells following Cm infection. Therefore, the protective role of early production of IL-17 and IFN*γ* by V*γ*4+ T and V*γ*1+ T cells is not essential but supplementary in clearance of Chlamydia. In our present study using Cm infection model, it was found that V*γ*4+ T cells were the major source of IL-17 in the early stage, and V*γ*1+ T cells did not secrete IL-17. Similarly, *Listeria monocytogenes* also induces *γδ* T cells, especially V*γ*4+ T and V*γ*6+ T cells, and secretes IL-17 in infected liver, but more than 60 percent of the IL-17 are produced by V*γ*6+ T cell, which have fast kinetic response characteristics [26]. However, the chronic granulomatous disease leads to unrestrained V*γ*1+ *γδ* T-cell reactivity which dominantly produces IL-17. Furthermore, with anti-CD3 antibody and virus-LPS stimulation in vitro, V*γ*1+ T cells dramatically produced IL-17, while only IL-10+ V*γ*4+ T cells existed [38]. Unlike Th17 cells, the subsets of IL-17+ *γδ* T cell in varieties of pathogen infections are not always the same pattern, while these data suggest increased numbers of *γδ* T cells with cytokine-producing potential during immune response; any role for *γδ* T cell-derived cytokines in various model remains to be defined.

Notably, there are still a small number of IL-17-producing-V*γ*4-*γδ* T cell subsets which is not identified during Cm infection. Interestingly, in our model, V*γ*6+ cells also present to proliferate following the Cm infection at the middle stage, which might be an important IL-17-producing cell after the early infection stage. IL-17+ V*γ*6+ T cells promote cancer cell growth by mobilizing peritoneal macrophages in the mice model of ovarian cancer [39]. In *Listeria monocytogenes*, more than 60 percent of the IL-17 are produced by V*γ*6+ T cell in infected liver, which have fast kinetic response characteristics [26]. In this study, we did not focus on V*γ*6+ T cell because it is reported that V*γ*6+ cells are the major *γδ* T cell population in reproductive tract but not in lungs [40]. But it still can be speculated that IFN*γ* and IL-17 may be partially secreted by V*γ*6+ T cells apart from V*γ*1+ T and V*γ*4+ T cells during Cm infection.

In conclusion, our data show that V*γ*1+ T and V*γ*4+ T cells are the major proliferative cell subsets of *γδ* T cell during Cm lung infection in mice. Moreover, V*γ*4+ T cells are the major IL-17 and IFN*γ*-producing *γδ* T cell subsets at the early period of Cm lung infection. The findings in the present study provide new insights into the mechanisms bridging innate and adaptive immunity during lung chlamydial infections, which may have implications in developing effective chlamydial vaccines and in the understanding of host defense mechanisms in other lung infections.
